# Around the Hospitals

**Published:** 1893-04-08

**Authors:** 


### Around the Hospitals.

**The Hospital for Diseases of the Throat,** Golden Square.—Sir F. Dixon-Hartland, M.P., presided at the annual meeting of this institution. Donations and subscriptions during the past year show a falling off, and an appeal is made for more assistance, as not only is it necessary to obtain a larger income for the general purposes of the hospital, but an extension is urgently needed, for which £5,000 is asked.

**The City of London Hospital** presents a good report for the past year. In regard to patients there has not been a single casualty, although the number of admissions has exceeded that of previous years. In the out-patients' department 1,536 women were delivered in their own homes. A large number of pupils have been trained during the year, and this branch of the work showing a steady increase, it is proposed to provide further accommodation. In June the hospital will be closed for necessary painting and cleaning. The bed at the Mothers' Seaside Convalescent Home has proved most beneficial, and the Samaritan Society, which has done good work, shows a balance in hand. Financially, the hospital is in a good condition, and it is hoped that this prosperity will continue through the continued efforts of friends on its behalf.

**North London Consumption Hospital.**—We are glad to learn that this hospital has not been forgotten this year in the matter of legacies, although annual subscriptions, still urgently needed, show a falling off instead of an increase. The useful character of the hospital has been testified to by liberal contributions towards the building fund, whereby the work will be usefully extended. Premises have been acquired in Fitzroy Square to serve as central offices and out-patients' department. The new central block at the hospital itself is progressing apace, and the interior will probably be finished by midsummer. The addition is a most welcome and necessary one. It is not proposed to occupy the whole of the accommodation at once, as the outlay required for this would be greater than the committee feel justified in sanctioning immediately. This new block will contain, besides wards for thirty patients, entrance hall, patients' dining hall, and nurses' and staff accommodation.

**The Queen's Hospital, Birmingham.**—Three matters of interest were discussed at the recent annual meeting of this hospital—the increase in the number of beds, the rearrangement of the casualty, and the question of finance. The maximum number of beds in the hospital is now occupied, and this naturally calls for increased support. Since the last meeting of the hospital a registration fee of one shilling has been required of casualty patients. This course is generally approved of, being framed to prevent abuse of the charity. It was stated at the meeting that where the patient was the bread-winner of the family, the fee was not insisted upon. In regard to finance, it was stated that the funds of the hospital are at a low ebb, and if the proposal to supply patients with tea, sugar, and butter is carried out, a still greater difficulty will be raised. The institution has unreasonably heavy calls upon it—Walsall, Coventry, Bromsgrove, and Halesowen send patients costing upwards of £285 per annum, whilst they subscribe but £21 in return.

